# Comment on “CCN1 secreted by human adipose‐derived stem cells enhances wound healing and promotes angiogenesis through activating the AKT signalling pathway”

**DOI:** 10.1111/iwj.14126

**Published:** 2023-03-28

**Authors:** Zheng‐Dong Yuan, Jun‐Jie Wu

**Affiliations:** ^1^ Wuxi Institute of integrated traditional Chinese and Western Medicine The Affiliated Hospital of Jiangnan University Wuxi China

Dear Editors,

We read this article written by Yi et al. titled “CCN1 secreted by human adipose‐derived stem cells enhances wound healing and promotes angiogenesis through activating the AKT signalling pathway”.^1^ This study investigates the role of the cellular communication network factor 1 (CCN1) secreted by human adipose‐derived stem cells (hADSCs) and suggests that it can play a crucial role in promoting skin wound healing. This finding is very important, as it suggests an alternative method for healing wounds. However, the article in our view has some limitations.

First, the sample size of five mice in each group is relatively small, which might limit the accuracy and the credibility of the conclusion. Therefore, it's necessary to expand the number of the sample. As a contrast, another research where sixteen mice in each group were included might be more reliable.^2^

Second, we observed that in this study, conditioned medium (CM) was directly injected into four sites in the wound edge and bed, and whether CCN1 in CM can be fully absorbed by the wound tissues after administration is a question worth discussing. Because in another practice, the injection of drugs results in skin popping which causes abscesses, infections, and other life‐threatening conditions.^3^

The last but not the least, wound healing is a complex process. It has three major overlapping phases: inflammation, formation of new tissues, and remodelling. The evaluation indicators of wound healing are not only re‐epithelialization and angiogenesis but also collagen A deposition and regeneration of accessory organs such as hair follicles.^4^ More assessment of wound healing is needed in this paper.

In summary, these data show that the CCN1 from hADSCs can promote wound healing, which may be a promising alternative for the wound healing.

## CONFLICT OF INTEREST STATEMENT

The authors declare no conflicts of interest.

## Data Availability

Data sharing not applicable to this article as no datasets were generated or analysed during the current study.
